# Naturally Occurring Triggers that Induce Apoptosis-Like Programmed Cell Death in *Plasmodium berghei* Ookinetes

**DOI:** 10.1371/journal.pone.0012634

**Published:** 2010-09-09

**Authors:** Medhat Ali, Ebtesam M. Al-Olayan, Steven Lewis, Holly Matthews, Hilary Hurd

**Affiliations:** 1 School of Life Sciences, Keele University, Keele, United Kingdom; 2 Department of Zoology, Ain Shams University, Cairo, Egypt; 3 Department of Zoology, King Saud University, Riyadh, Saudi Arabia; INSERM U1016, Institut Cochin, France

## Abstract

Several protozoan parasites have been shown to undergo a form of programmed cell death that exhibits morphological features associated with metazoan apoptosis. These include the rodent malaria parasite, *Plasmodium berghei*. Malaria zygotes develop in the mosquito midgut lumen, forming motile ookinetes. Up to 50% of these exhibit phenotypic markers of apoptosis; as do those grown in culture. We hypothesised that naturally occurring signals induce many ookinetes to undergo apoptosis before midgut traversal. To determine whether nitric oxide and reactive oxygen species act as such triggers, ookinetes were cultured with donors of these molecules. Exposure to the nitric oxide donor SNP induced a significant increase in ookinetes with condensed nuclear chromatin, activated caspase-like molecules and translocation of phosphatidylserine that was dose and time related. Results from an assay that detects the potential-dependent accumulation of aggregates of JC-1 in mitochondria suggested that nitric oxide does not operate via loss of mitochondrial membrane potential. L-DOPA (reactive oxygen species donor) also caused apoptosis in a dose and time dependent manner. Removal of white blood cells significantly decreased ookinetes exhibiting a marker of apoptosis in vitro. Inhibition of the activity of nitric oxide synthase in the mosquito midgut epithelium using L-NAME significantly decreased the proportion of apoptotic ookinetes and increased the number of oocysts that developed. Introduction of a nitric oxide donor into the blood meal had no effect on mosquito longevity but did reduce prevalence and intensity of infection. Thus, nitric oxide and reactive oxygen species are triggers of apoptosis in *Plasmodium* ookinetes. They occur naturally in the mosquito midgut lumen, sourced from infected blood and mosquito tissue. Up regulation of mosquito nitric oxide synthase activity has potential as a transmission blocking strategy.

## Introduction

There is a well established body of literature that recognizes programmed cell death (PCD) as a signal dependent, active process of cell suicide that occurs in unicellular eukaryotes as well as metazoans. Its origin predates the evolution of multicellularity and may have arisen during the endosymbiotic incorporation of the mitochondrial precursor into the ancestral eukaryote [1]–[4]. Galluzzi and colleagues speculate that many of the cell death regulators of metazoan cells may originally have been involved in stress regulation [5].

Three types of PCD have been well characterized in metazoans, including type 1 PCD, or apoptosis [6], [7] however, it is now recognized that overlapping pathways leading to different types of PCD death exist, and more complex nomenclature has been suggested [8]. Despite this, new recommendations concerning death-related terminology have recently been published and the used of just the three terms, necrosis, apoptosis and autophagic cell death are recommended [9]. Most of the features that are signatures of apoptosis in metazoans are also present in unicellular eukaryotes, including loss of mitochondrial outer membrane permeability (MOMP), nuclear chromatin condensation, cytochrome c release, DNA fragmentation and translocation of phosphatidylserine (PS) to the outer cell membrane (reviewed in [10], [11]). Despite this, much of the apoptotic molecular machinery common to multicellular eukaryotes is absent, and different pathways may be leading to the same endpoint [11]–[13].

Markers of apoptosis have been described in both the vertebrate and vector stages of the malaria parasite [14]–[17] although, as discussed by Arambage and colleagues [18], conflicting results have emerged from different laboratories, particularly regarding apoptosis in erythrocytic stages. In the mosquito midgut lumen, motile zygotes or ookinetes are formed around 18 h post-infection and move out of the blood meal bolus before traversing the midgut epithelium to form oocysts beneath the basal lamina. Within the midgut lumen of *Anopheles stephensi,* over 50% of ookinetes of the rodent malaria, *Plasmodium berghei*, exhibit markers of apoptosis by 24 h post-infection [16].This cell suicide may help to explain the huge loss of parasites between the ookinete and oocyst stage that has been reported during many *Plasmodium*/mosquito associations [19].

Cell suicide in protists can be triggered by various extra and intracellular events including nutrient deprivation, heat shock, drug treatment and the presence of nitric oxide (NO), reactive nitrogen species (RNS) and reactive oxygen species (ROS) (see for example reviews [11]–[13]). Many investigations of parasitic protists are conducted *in vitro* and apoptosis is induced experimentally by one or other of these triggers. However, protists have been shown to exhibit markers of apoptosis *in vivo*, without experimental manipulation, and apoptosis has been suggested to play an important role in the life history of some unicellular parasite populations. For example, in trypanosomatids it is thought to act as a mechanism to control population numbers and eliminate particular developmental stages [11], [20]–[23]. Extrinsic inducers of apoptosis in parasitic protozoans must therefore occur *in vivo* and would be expected to be found in the mosquito midgut lumen following an infective blood meal.

The mosquito midgut lumen is an environment that becomes increasing hostile as the blood meal is digested; in part this is due to the presence of NO, RNS and ROS. Peterson and colleagues [24] suggest that haemoglobin, present in the blood meal, catalyses the synthesis of NO metabolites in a reducing environment. An additional source of NO comes from the mosquito as nitric oxide synthase (NOS), present in midgut epithelial cells, catalyses the production of NO during the oxidative deamination of L-arginine to L-citrulline. Inducible *NOS* expression is upregulated in mosquito midgut epithelial cells in response to malaria infection [25], [26] and induction of *NOS* is proportional to the intensity of infection [27]. Herrera-Oritz and colleagues [28] also reported that NO is produced in midguts of *Anopheles pseudopunctipennis* cultured with *P. berghei.* In addition, NOS has been detected within midgut cells that have been invaded by ookinetes [29], [30].

NO, or its reactive derivatives, play a part in the immunological reaction of host defence against numerous pathogens [31]. They have been identified as important cytotoxic effector molecules in a variety of parasitic infections of vertebrates, including schistosomiasis, leishmaniasis and malaria infection [32]. NO has been shown to have a cytotoxic or cytostatic effect on erythrocyte stages of *Plasmodium*, depending on concentration [33]–[35] and Rockett and colleagues found that *S*-nitrosothiols displayed a far greater toxicity to *P. falciparum* than nitrate which, in turn, was more effective than nitrite [36]. The availability of NO for *Plasmodium* killing in the vertebrate circulatory system has been questioned [37], nevertheless, it has indisputable effects on malaria transmission [38]: killing gametocytes [39] and ookinetes [40] and nitrate concentration increases in the gut of Plasmodium-infected mosquitoes [41]. In the mosquito, inhibition of *NOS* activity has been shown to increase the number of oocysts developing on the midgut [26].

Herrera-Ortiz *et al* [28] found that ookinetes incubated with the NO donor, sodium nitroprusside (SNP) or the ROS generator, L-DOPA, significantly decreased viability and that a combination of both donors was synergistic. Similarly, L-DOPA dependent superoxide anion (O_2_^-^), produced in the haemolymph and midgut of *Anopheles albimanus*, is toxic to ookinetes [40]. L-DOPA, also caused loss of *P. berghei* gametocyte infectivity [42]. In addition to RNS, ROS are also generated within the midgut cells of *An. stephensi* during ookinete invasion [43]. We hypothesized that these toxic molecules may be naturally occurring extrinsic triggers of apoptosis in *P. berghei* ookinetes.

NO has been shown to create nitrosative stress which can promote apoptosis by the activation of mitochondrial apoptotic pathways [44] and mitochondrially derived ROS have also been associated with the initiation phase of apoptosis, acting as mediators for different signal transduction pathways. A role for ROS in ancient redox –sensitive pathways leading to apoptosis-like PCD has also been proposed and both RNS and ROS have been shown to act as triggers for apoptosis in *Toxoplasma gondii* [45] and kinetoplastid parasites of the genera *Trypanosoma and Leishmania* [46]–[49].

The purpose of this study was to investigate the hypothesis that NO and ROS cause the death of *P. berghei* ookinetes by an apoptosis-like (hereafter referred to as apoptosis) process. Investigations were performed *in vitro* and ookinetes examined for several markers of apoptosis following exposure to donors of NO and ROS. Experiments were then designed to determine whether natural sources of NO in the blood meal and mosquito midgut could also induce the display of markers of apoptosis *in vivo*. The results demonstrated that NO, or its derivatives, do trigger apoptosis in ookinetes *in vitro* and support the hypothesis that NOS activity is linked to the induction of apoptosis in *P. berghei* ookinetes whist they are in the mosquito midgut lumen. The role of ROS in inducing apoptosis is less marked, although we do demonstrate that it is toxic to ookinetes *in vitro* and causes the display of markers of apoptosis in a dose and time dependent manner.

## Results

### The effect of white blood cells on the induction of apoptosis

White blood cells (WBCs) contain NOS and thus represent a source of NO in the blood. In order to determine whether the removal of these cells from gametocytaemic blood prior to culture affected the proportion of ookinetes that exhibited signs of chromatin condensation, a comparison was made between blood with and without WBCs. Exposure to WBCs caused a small, but overall significant, increase in the proportion of ookinetes with acridine orange staining (depicting condensed chromatin) when maintained in Schneider's medium for 0.25 h, 3 h and 6 h respectively (∼5.5, 4 and 1% increase; F_1,35_ = 4.76; *P<*0.05) (see supplementary Figure S1). The remainder of ookinete incubations in this study was performed in the presence of WBCs.

### The effect of NO donors on ookinetes *in vitro*

**Figure 1:**
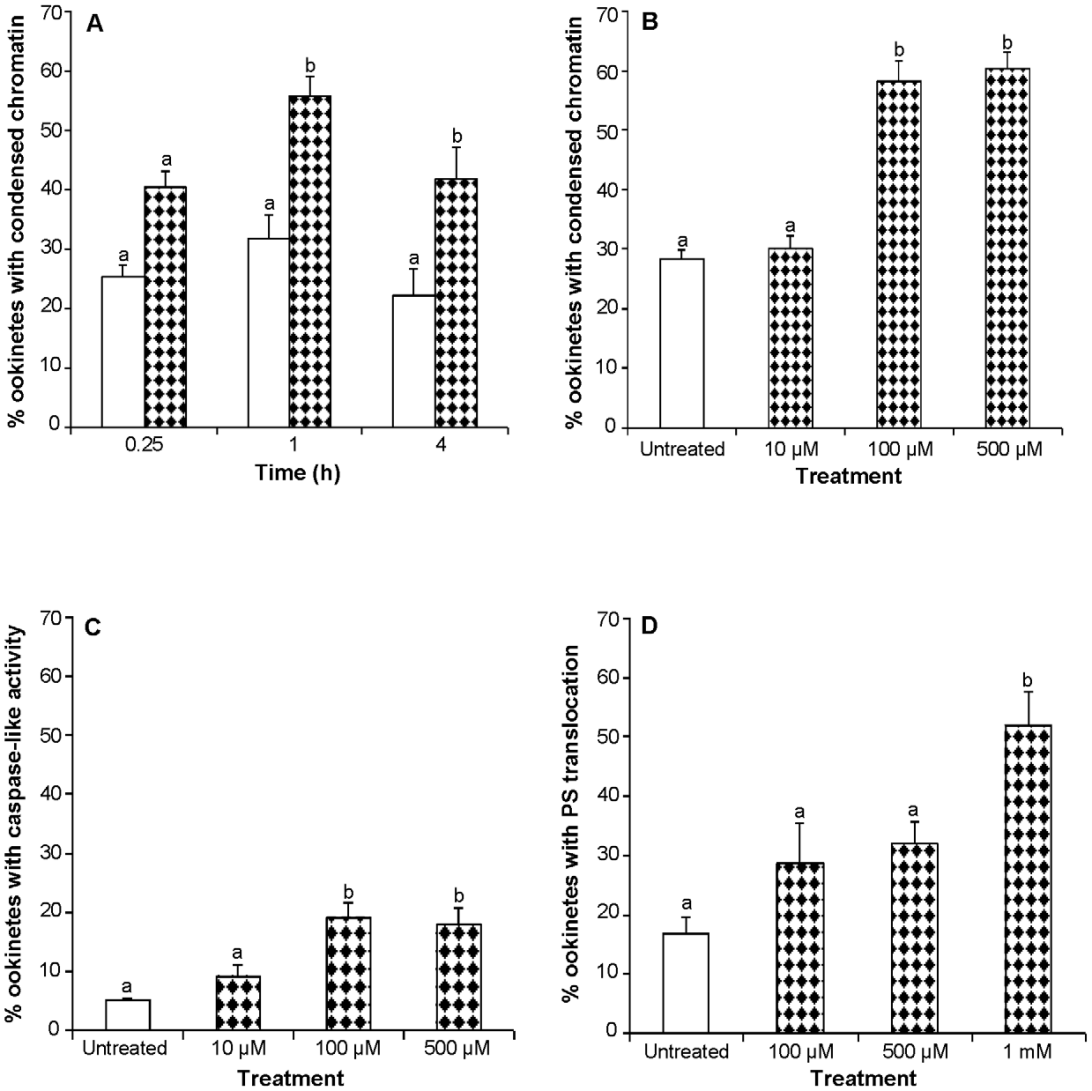
The effect of nitric oxide donors on the induction of apoptosis in *P. berghei* ookinetes. Ookinetes were incubated in RPMI with or without the addition of SNP at different concentrations or for different time periods and then examined for the presence of different markers of apoptosis. **A**:The effect of 2 mM SNP on the proportion of ookinetes expressing a marker of apoptosis; data are means of 3 experiments (n = 3) with 300 ookinetes examined in each experiment. **B,C**: The effect of different concentrations of SNP following 1 h incubation; n = 3, with 100–150 ookinetes examined per experiment). **D**: The effect of different concentrations of SNP on ookinetes following 4 h incubation; n = 3, with 25–50 ookinetes examined in each experiment. Apoptosis markers examined were condensed nuclear chromatin (**A**, **B**), activated caspase-like molecules (**C**), and PS translocation without loss of membrane integrity (**D**). Error bars represent standard error of the mean (SEM). Bars with different letters are significantly different. White bars represent controls and patterned bars represent treated ookinetes.

Newly formed ookinetes were exposed to 2 mM sodium nitroprusside (SNP) for 0.25 h, 1 h or 4 h and the percentage of ookinetes displaying condensed chromatin was detected using acridine orange staining. An overall significant increase in acridine orange-positive ookinetes occurred (F_1,53_ = 40.29; *P<*0.001) and pair wise comparisons showed that this increase was significant after 1 h (∼25% increase *P*<0.001) and 4 h (∼20%, *P*<0.01) incubations (see Figure 1A). Ookinetes were also cultured for 24 h but by this time the proportion of parasites with condensed chromatin in the control group had risen to over 70% and no significant differences were detected between control and treated groups (data not shown). NO thus accelerated the induction of apoptosis in a large proportion of the ookinete population. An investigation into the effect of different concentrations of SNP showed that the effect was dose dependent; although 10 µM SNP did not induce additional ookinetes to undergo chromatin condensation, 100 µM SNP caused a significant increase of 30% (*P*<0.001) and 500 µM SNP caused an increase of 32% (*P*<0.001) (Figure 1B). Further experiments incubating ookinetes with 1 mM, 2 mM and 4 mM SNP for 1 h and 4 h all showed significant increases in ookinetes displaying this marker for apoptosis compared with untreated groups at the same time points. However, in no case were all ookinetes induced to undergo apoptosis; between 20 and 30% never displayed chromatin condensation, even after 24 h incubation with SNP (data not shown).

Two additional markers for apoptosis-like death, the presence of activated caspase-like molecules and the translocation of PS to the outer membrane were monitored after SNP treatment. As the percentage of untreated ookinetes with apoptosis markers varied greatly between experiments, fold increases in the expression of each marker were calculated to facilitate comparison between experiments (Table 1). A similar pattern to chromatin condensation was seen with an overall significant increase in the percentage of ookinetes expressing active caspase-like molecules in SNP-treated ookinetes compared to that of untreated ookinetes (F_3,23_ = 9.6; *P*<0.001). This was caused by incubation with 100 µM and 500 µM SNP but not with the lower concentration of 10 µM SNP (see Figure 1C). Although the proportion of untreated ookinetes displaying caspase-like activity was much smaller than those displaying acridine orange staining in the previous experiment the proportion of apoptotic ookinetes in the untreated group was lower in these experiments and a similar fold increase in this marker occurred upon treatment (see Table 1). Examination of ookinetes for PS translocation revealed that incubation with SNP also caused a significant increase in this marker of apoptosis (F_3,19_ = 8.93; *P<*0.01) but that higher concentrations (1 mM) and a longer incubation period (4 h) were required for the proportion of annexin positive ookinetes (that did not have compromised membranes) to be significantly higher than untreated parasites (Figure 1D and Table 1). The yield of parasites available for this latter series of experiments was lower than the previous ones and thus the number of ookinetes examined was smaller.

**Table 1:** The fold-increase in the percentage of apoptotic ookinetes treated with SNP, relative to untreated ookinetes.

Apoptotic marker	Concentration	Fold-increase
Condensed chromatin	10 µM	1.06
	100 µM	2.08**
Activated caspase-like molecules	10 µM	1.72
	100 µM	3.86**
	500 µM	3.72**
PS translocation after 1 h	100 µM	2.17
	500 µM	2.18
	1 mM	2.33
PS translocation after 4 h	100 µM	1.88
	500 µM	2.11
	1 mM	3.85*

Apoptotic ookinetes were identified using the following markers; condensed chromatin, activated caspase-like molecules or phosphatidyl (PS) translocation to the outer cell membrane. Significant difference from controls, *P<0.05 and ** *P*<0.001.

**Figure 2:**
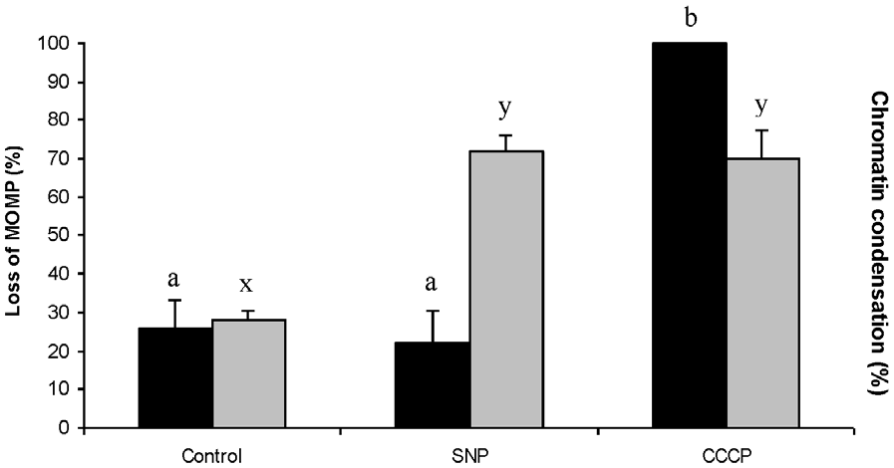
Loss of mitochondrial membrane potential and the induction of apoptosis in *P. berghei* ookinetes. Ookinetes were maintained in RPMI alone (controls) or incubated with 100 µM SNP for 2 h or with CCCP for 30 min prior to examination for loss of mitochondrial membrane potential (MOMP) (black bars) or the presence of nuclear chromatin condensation (grey bars). Error bars represent standard error of the mean (SEM), n = 4 with 50–100 ookinetes examined in each experiment. Bars with different letters are significantly different.

Ookinetes that had been collected from gametocytaemic-blood following 18 h of culture were used to determine whether the execution pathway of NO-induced apoptosis involved the mitochondrion. Ookinetes were incubated for 2 h with 100 µM SNP and then examined for loss of MOMP using a JC-1 assay kit. Approximately 30% of untreated ookinetes exhibited loss of MOMP and a similar proportion to this contained nuclei with condensed chromatin (Figure 2). Further ookinetes were, instead, incubated with carbonyl cyanide 3-chlorophenylhydrazone (CCCP), which causes loss of MOMP, and compared with untreated samples and SNP treated samples. All the ookinetes in samples treated with CCCP had lost MOMP (Kruskal-Wallis *p* = 0.008), however SNP treatment did not induce further loss of MOMP. Overall, both SNP and CCCP treatments caused a significant increase in the proportion of ookinetes displaying condensed chromatin (F_2.9_ = 18.93; *p* = 0.001), and each group differed significantly from the untreated parasites (Figure 2). These data suggest expression of this apoptosis marker in ookinetes may occur via a pathway involving loss of mitochondrial membrane potential (Δ ψ_m_), but that NO-induced apoptosis does not proceed in this way.

In order to confirm that the induction of apoptosis seen following incubation with SNP was due to the NO produced by this donor some supplementary investigations were performed using two other donors to deliver NO to ookinetes in culture. It took 500 µM S-Nitroso-N-acetyl-DL-penicillamine (SNAP) to induced a significant increase in the proportion of ookinetes displaying caspase-like activity after 1 h incubation (*P*<0.01). However S-nitroglutathione (SNOG) only induced a significant increase in the proportion with condensed chromatin at a concentration of 2 mM after incubation for 6 h (*P*<0.001) (Supplementary Figure S2A and B).

Measurement of the nitrite produced by the three donors when dissolved in solutions of supplemented RPMI revealed large differences in the speed and amount of release, with SNAP producing more nitrite that the other two donors during short incubation times whereas SNOG produced much more after 24 h (Supplementary Figure S3). Some of the nitrite produced by these donors may have been oxidized to nitrate during these incubation periods but this would not be detected by the Griess reaction use in this study.

### The effect of a nitric oxide synthase inhibitor on apoptotic ookinetes *in vivo*

**Figure 3:**
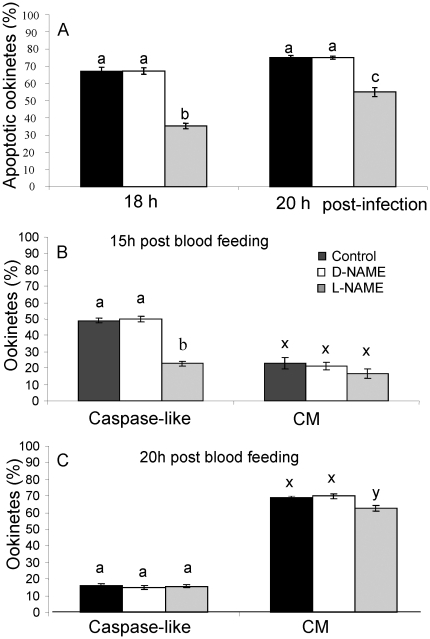
The effect of a nitric oxide synthase inhibitor on the expression of apoptosis markers by ookinetes *in vivo*. *An. stephensi* were fed on blood containing *P. berghei* gametocytes (black bars) with the addition of L-NAME (grey bars) or the inactive isomer D-NAME (white bars). **A:** The proportion of ookinetes with condensed nuclear chromatin at 18 h and 20 h post-infection. **B**, **C**:The proportion of ookinetes expressing caspase-like activity or with compromised membranes (CM) 15 h and 20 h post-infection respectively. Error bars represent standard error of the mean (SEM). Bars with different letters are significantly different.

In addition to WBCs in the blood meal, the mosquito midgut epithelium is a source of NO that could induce apoptosis in developing ookinetes. To test this hypothesis, mosquitoes were fed a NOS inhibitor, N*w*-nitro-L-arginine methyl ester (L-NAME), the inactive isomer, N*w*-nitro-D-arginine methyl ester (D-NAME), or infective blood meal alone. Analysis of the number of ookinetes in the midgut lumen that were exhibiting condensed chromatin in the presence of a NOS inhibitor or the inactive isomer indicated significant differences due to treatments, (*F*_2,12_ = 123.86, *P*<0.001), time post-infection, (*F*_1,12_ = 63.379, *P*<0.001), and treatments by time interaction (*F*_2,12_ = 7.05796, *P*<0.01). Specifically, the addition of L-NAME to the blood meal caused a significant reduction in the proportion of ookinetes exhibiting condensed chromatin (*P*<0.001) compared to those exposed to control treatments at 18 h and 20 h post-blood feeding. Additionally, significantly more L-NAME treated ookinetes were undergoing apoptosis at 20 h than 18 h post-infection (*P<*0.001) but this increase was not detected in the control treatments, suggesting that inhibition of NOS activity had delayed the induction of apoptosis (Figure 3A).

In the anticipation that activation of cysteine proteases may occur upstream of chromatin condensation we monitored the effect of inhibition of NOS activity earlier, at 15 post blood- feeding. Approximately 50% of the 200 ookinetes examined stained positive for caspase-like activity in untreated and D-NAME treated mosquitoes by 15 h post-feeding and a further 20% were permeable to propidium iodide (PI) (which may have obscured staining for caspase-like activity) and thus classed as dead (Figure 3B). An overall significant change in these proportions had occurred by 20 h post-infection (F_5,24_ = 573.2 *P<*0.001 (caspase) F_5,24_ = 47.8 *P<*0.001(compromised membranes)) (Figure 3C) with the proportion of caspase positive ookinetes with intact membranes declining, those with compromised membranes increasing and lack of visible staining for caspase-like activity increasing. Intermediate values were recorded for each parameter at 18 h (data not shown). The addition of L-NAME to a blood meal significantly decreased the proportion of ookinetes with activated caspase-like molecules in their cytoplasm at 15 h (*P<*0.001) whereas D-NAME had no effect (*P*>0.1) but by 20 h there was no significant difference between NOS-inhibitor treated ookinetes and the control (*P>*1.0) or D-NAME (*P>*0.99). However, significantly fewer L-NAME treated ookinetes had compromised plasma membranes (*P<*0.02) at this time (Figure 3C). These data again suggest that inhibition of NOS activity gave some initial protection against apoptosis.

Further experiments demonstrated that the addition of the NOS inhibitor L-NAME to the blood meal significantly increased the number of *P. berghei* oocysts developing on the midgut of *An. stephensi* 12 days post-infection compared with those in the D-NAME treated groups and untreated mosquitoes (see Table 2). No differences in mosquito survivorship were observed across the treatments (data not shown).

**Table 2:** The effect of nitric oxide synthase activity on the number of oocysts developing in the midgut of *An. stephensi* mosquitoes.

Treatment	Mosquitoes surviving after day 12 post-infection	†Oocysts/gut (± SEM)	*P*-value
Control	48/50	44.60 (1.62)	NS
D-NAME	46/50	40.37 (1.54)	NS
L-NAME	49/50	85.91 (5.81)	0.001*

† Oocysts developing on the midgut were counted on day 12 post-infection.

*Significantly different from the control, untreated group.

### The effect of an NO donor on mosquito longevity and ookinete development *in vivo*

**Figure 4:**
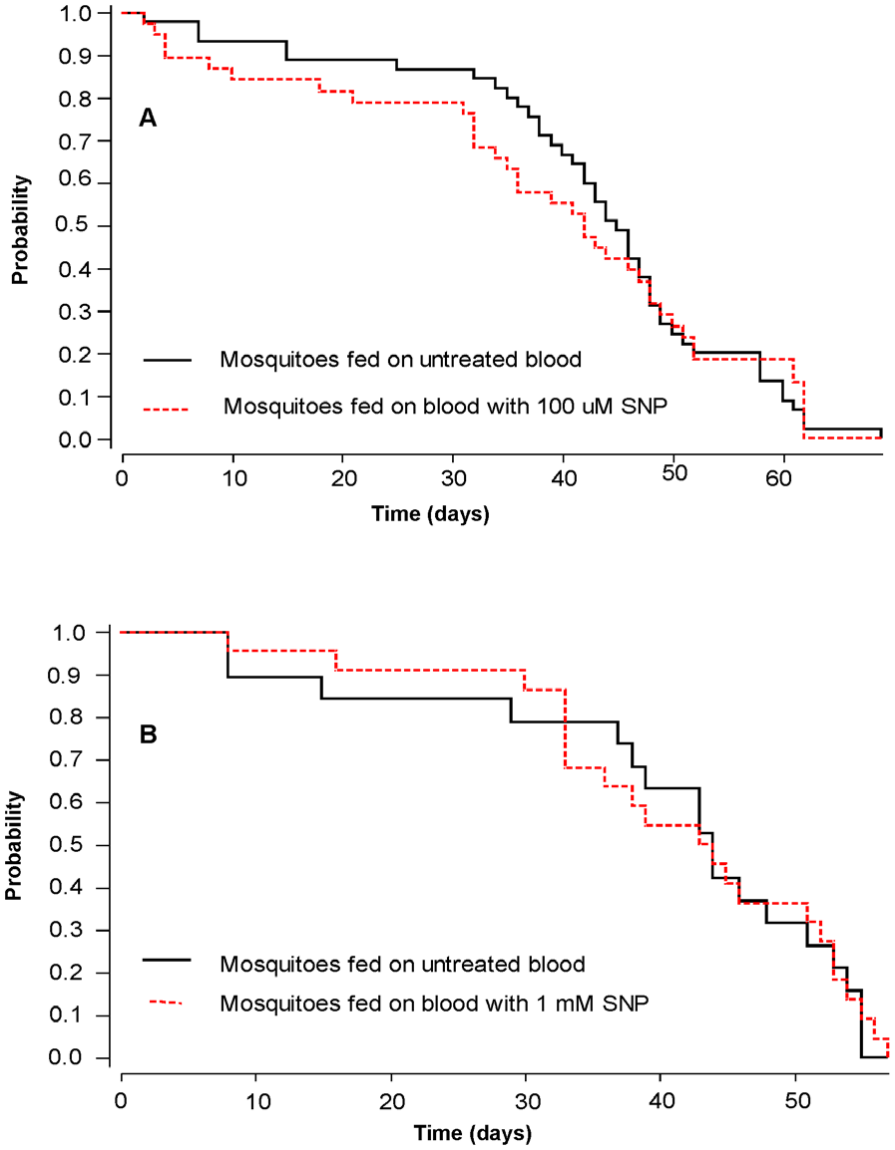
The effect of SNP on the survivorship of *P. berghei*-infected mosquitoes. Four groups of 38–45 mosquitoes were fed on *P. berghei*-infected mouse blood (solid black lines, 2 groups), or *P. berghei*- infected mouse blood with the addition of 100 µM SNP (**A**) or 1 mM SNP (**B**) (dotted red lines). Mosquito deaths were recorded daily.

An assessment was made of the feasibility of enhancing the natural production of NO by the mosquito as a means of killing ookinetes *in vivo* without affecting mosquito fitness. The effect on mosquito longevity of administration of the NO donor SNP with an infective blood meal was assessed. Neither 100 µM nor 1 mM SNP caused a significant change in the probability of survival compared with untreated mosquitoes (Wilcoxon analyses: 100 µM, *P* = 0.9 and 1 mM (*P* = 0.98) (Figure 4A and 4B), demonstrating that one blood meal containing elevated levels of NO did not affect longevity.

**Figure 5:**
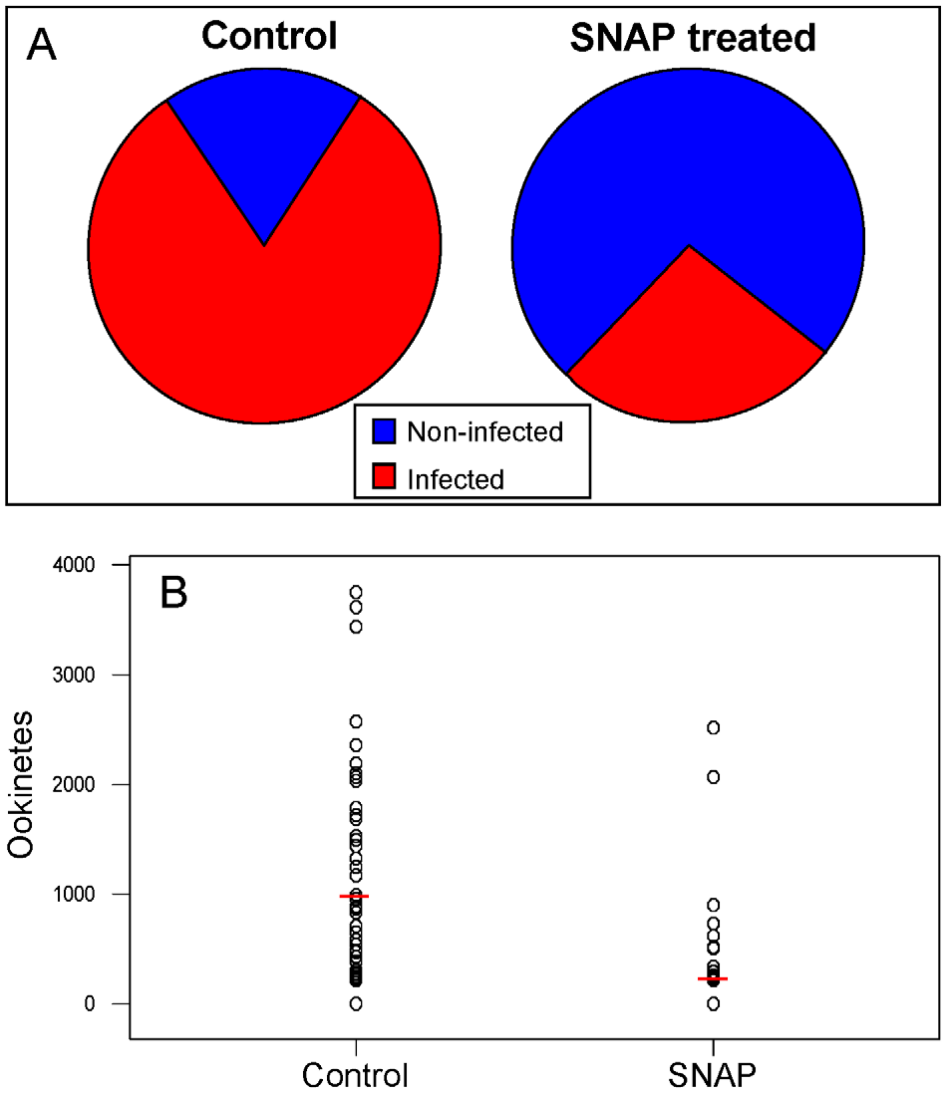
The effect of SNAP on the prevalence and intensity of *P. berghei* infection in *An. gambiae.* Mosquitoes were fed on gametocytaemic mouse blood alone or on blood containing 100 µM SNAP and midguts examined 16–18 h post-infection. **A:** Prevalence of ookinete infections (mean of 5 experiments). **B:** Intensity of infection (the number of ookinetes recovered from each midgut; infected blood, n = 60, infected blood with SNAP, n = 55.

Additional NO in the blood meal did, however, have an effect on the prevalence of mosquito infections which fell significantly (P<0.01) when mosquitoes fed on an infective blood meal containing 100 µM SNAP (Figure 5A) and mean numbers of ookinetes found in the midgut lumen dropped from 969±120 to 207±62 (P<0.001) (See Figure 5B).

### The effect of reactive oxygen species on ookinetes

**Figure 6:**
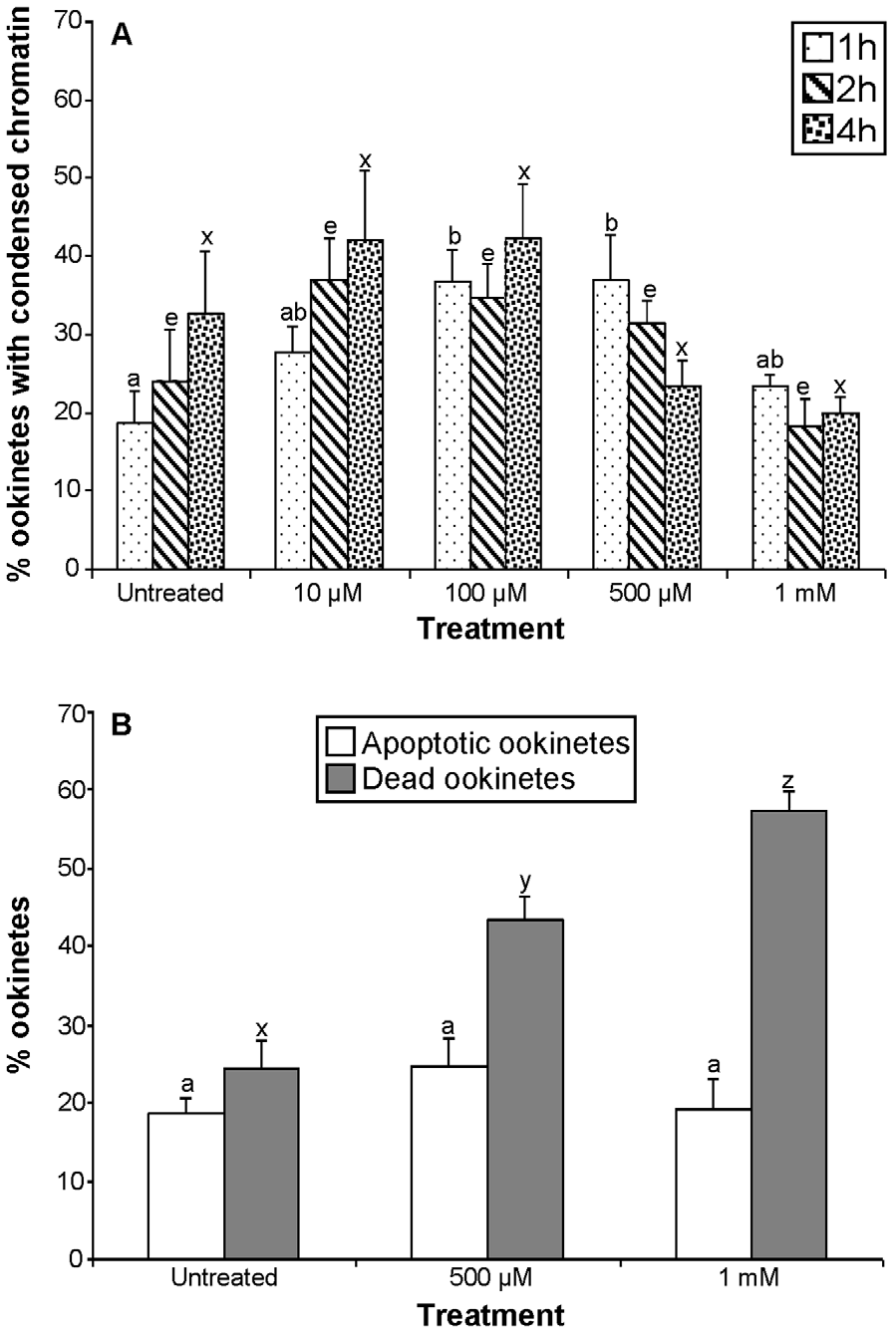
The effect of L-DOPA on the induction of apoptosis *in P. berghei* ookinetes *in vitro.* **A:** Ookinetes were incubated in supplemented RPMI containing different concentration of L-DOPA for 1 h (bars with small dots), 2 h (bars with strips) and 4 h (bars with large dots) and the proportion containing condensed nuclear chromatin was calculated. **B:** Ookinetes were incubated with L-DOPA for 4 h and those with compromised membranes (dead ookinetes) were identified by staining with erythrosin B (grey bars), while ookinetes showing chromatin condensation were identified with acridine orange (white bars). Bars are a mean of 3 experiments (n = 3) with at least 50 ookinetes examined in each experiment. Error bars represent SEM.

The effect of L-DOPA on ookinetes was both dose and time dependent. Thus, compared to controls, the increase in ookinetes expressing chromatin condensation after 1, 2 and 4 h exposure to 10 µM L-DOPA was not significantly different (*P* = 0.9, *P* = 0.7 and *P* = 0.9, respectively), but concentrations of 100 and 500 µM L-DOPA applied for 1 h did significantly increase this apoptosis marker (*P<*0.05). However, at higher concentrations and longer incubation times the proportion of parasites with condensed chromatin was no greater than that of untreated samples (Figure 6A). Instead, the proportion of ookinetes with compromised membranes rose steadily with time and concentration of L-DOPA (Figure 6B). It should be noted that the increase in apoptotic ookinetes seen when treated with 500 µM L-DOPA for 4 h was not significant (Figure 6B).

**Figure 7:**
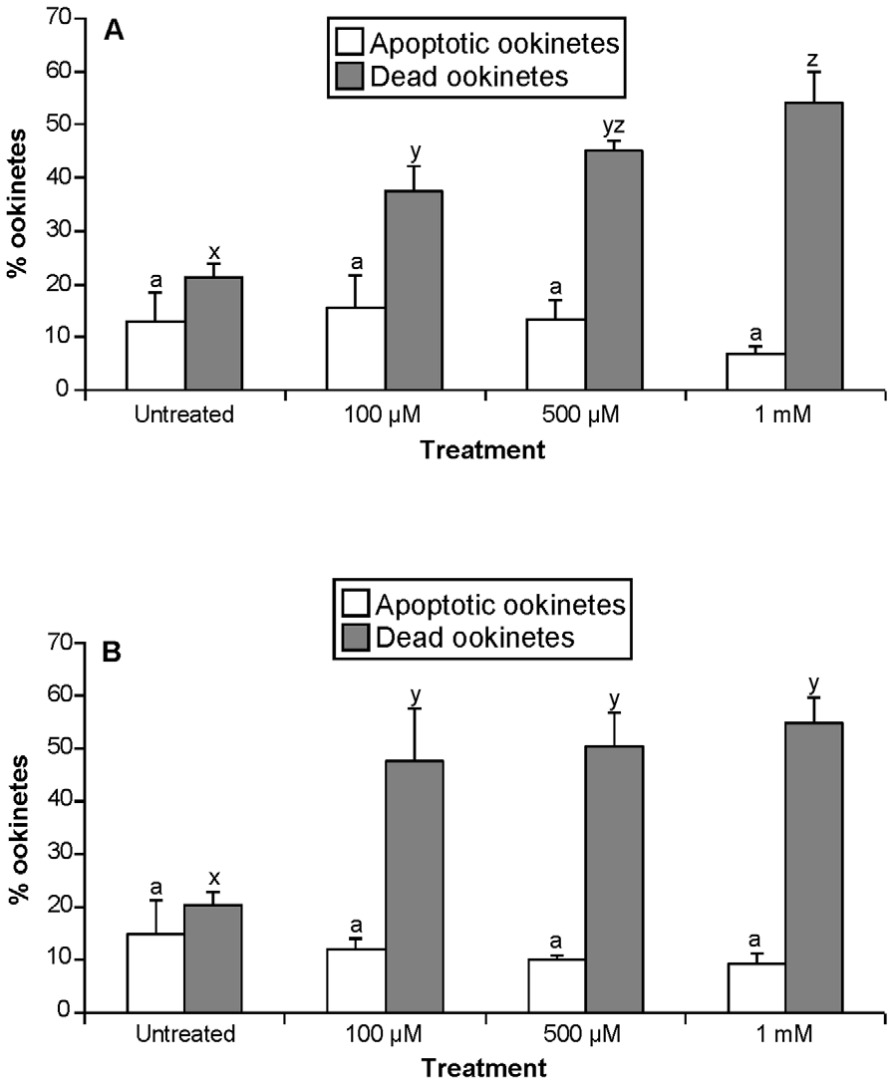
The effect of H_2_O_2_ on the induction of apoptosis of ookinetes i*n P. berghei* ookinetes *in vitro.* The effect of incubation with different concentrations of H_2_O_2_ for 1 h (**A**) or 4 h (**B**). Apoptotic ookinetes displaying chromatin condensation were detected with acridine orange (white bars); ookinetes with compromised membranes were detected with propidium iodide (grey bars). Bars represent means of 3 experiments and at least 25 ookinetes were counted per sample. Error bars represent SEM and bars with different letters are significantly different.

Another reactive oxygen species generator, H_2_O_2_, caused a significant loss of membrane integrity within 1 h of exposure to 100 µM, 500 µM or 1 mM concentrations (*P<*0.001) and a larger proportion of the population had compromised membranes when incubated with 100 µM, or 500 µM H_2_O_2_ for 4 h. However, no increase in the proportion of ookinetes displaying nuclear chromatin condensation occurred following 1 h or 4 h incubation with these concentrations of H_2_O_2_ (Figure 7A,B).

## Discussion

We previously reported, that a proportion of *P. berghei* ookinetes that develop *in vitro* and *in vivo* express markers of apoptosis without the addition of known inducers of apoptosis. [16]. The findings reported herein strongly support our hypothesis that NO and/or RNS act as inducers of apoptosis in *P. berghei* ookinetes *in vitro* and that this induction is dose and time dependent. We show that incubation with the NO donor SNP significantly increased the proportion of newly formed ookinetes exhibiting nuclear chromatin condensation, caspase-like activity and PS translocation when present at concentration that our experiments suggest would produce between 5–10 µM nitrite after 1 h. SNAP also induced caspase-like activity within 1 h at doses that generated low levels of nitrite (∼10 µM nitrite after 1 h). In contrast, higher concentrations and longer incubation periods were used to study the effect of the NO donor SNOG but the proportion of ookinetes displaying condensed chromatin did not significantly increase after 3 h exposure and a significant increase was only observed after 6 h incubation (expected nitrite production of ∼50 µM). It is possible that induction of apoptosis occurs within a narrow range of concentrations of NO or RNS and exposure to high levels of these compounds may cause death by other means such as necrosis. However, this does not explain why we observed this significant increase in chromatin condensation following 6 h incubation. It will be necessary to use several apoptosis markers to confirm that we were definitely observing apoptosis rather than necrosis when ookinetes are exposed to high levels of NO.

It would appear that L-DOPA-induction of the expression of apoptotic markers in ookinetes is highly sensitive to concentration and time of exposure. Very low concentrations of L-DOPA did not generate enough ROS to have any effect, whereas exposure to 1 mM or more resulted in loss of membrane integrity without markers of apoptosis being displayed. Although none of the concentrations of H_2_O_2_ used in this study induced apoptosis, it is possible that lower concentration would have done so. RNS and ROS have been identified as inducers of apoptosis in a variety of protozoans and their effect has been reported to be concentration dependent (see reviews [10]–[12]). For example, H_2_O_2_ at low concentrations (500 µM) caused *Tritrichomonas foetus* to undergo apoptosis-like death whereas at concentration of 8 mM necrotic death was observed [50].

It is worth noting the large variation in the proportion of apoptotic ookinetes observed at the beginning of different experiments. This could be an unavoidable consequence of having to use different mice as the source of infectious blood. We speculate that parameters such as intensity of asexual infection, number of gametocytes present or parameters such as NO or cytokine levels in the mouse plasma may be responsible for these differences.

The steady rise in the proportion of apoptotic ookinetes over time may be due to an increase in the level of RNS in the culture medium, or differences in susceptibility of individual parasites to NO/RNS (possibly associated with differences in ookinete age as development is asynchronous). It is known that, *in vitro*, late erythrocytic stages of *P. falciparum* are more vulnerable to the effects of NO produced by SNAP than the early (ring) stages and this effect is dose dependent [33]. In this and our previous studies, we note that approximately 20% of ookinetes remain viable even when exposed to inducers of apoptosis for long periods. Herrer-Ortiz and colleagues [28] also found that 28% of *P. berghei* ookinetes survive the oxidative stress generated by the combined action of L-DOPA and SNP. They suggested that this was due to the presence of adaptive, protective, molecules produced by ookinetes to fight mosquito defenses such as antioxidants. Even though these parasites originated from a single clone some individuals may be more vulnerable than others.

The demonstration that NO or ROS will induce apoptosis-like death of ookinetes *in vitro* did not, however, demonstrate that NO- or ROS-induced apoptosis has a role to play in the life history of the sporogonic stages of the malaria parasite. To support this proposal it was necessary to identify a potential source of NO, RNS or ROS in the mosquito midgut lumen and to demonstrate that redox generated reactions initiate PCD pathways in developing zygotes and ookinetes *in vivo*. Both the blood meal and mosquito tissue have been shown to be sources of these apoptosis triggers.

### Sources of nitric oxide

*Plasmodium* gametocytes are imbibed with a blood meal that, in addition to infected and uninfected erythrocytes, contains white blood cells, platelets and serum. Malaria infection elevates the level of cytokines such as TNF-α. IL-1 and IFN-γ that are known inducers of iNOS expression in mouse macrophages [51]. This induction acts synergistically with haemozoin in mouse [52], but not human, macrophages [53]. WBCs thus represent an endogenous source of NO. Cao *et al*. [38] reported that infected mouse serum, obtained 5 days after *P. yoelii* infection, blocks gametocyte infectivity to the mosquito via nitric oxide production and inactivation of gametocyte activity has been associated with the presence of WBCs. Furthermore, WBCs have been shown to remain active in the blood bolus for several hours and thus could contribute to killing mechanisms that operate in the mosquito midgut [39], [54]. It is likely that they are also active in gametocytaemic blood that is cultured to produce ookinetes *in vitro*. Previously, our data had shown that removal of WBCs from *in vitro* culture caused significant (13%), reduction in the number of apoptotic ookinetes (Al-Olayan and Hurd, unpublished). Here we confirm this finding. In both cases however the difference that the presence of WBCs made, to the proportion of ookinetes displaying markers of apoptosis was small. Therefore we note that the presence of WBCs is unlikely to account for induction of apoptosis in all the ookinetes in our untreated samples *in vitro* as this ranged from approximately 15 to 40% within 18 h of the culture of gametocytaemic blood, and rose steadily to a plateau that could be as high as 80% after 24 h. It is possible that the production of NO from WBCs might be low when cultured at 19°C (optimal developmental temperature for *P. berghei* ookinetes) and the NO they produce could play a more important role within mosquitoes, or in cultures kept at the higher temperatures conducive to the development of ookinetes of other species of malaria. However, within our system, we suggest there may be additional sources of NO, or other external triggers, that induce a large proportion of *P. berghei* ookinetes to undergo apoptosis.

Within the mosquito midgut, Luckhart and co-workers have identified inflammatory levels of reactive nitrogen intermediates in the blood meal [41] with nitrates and metal nitrosyls elevated following an infective blood meal Nitrogen oxides present in blood meals were measured using a nitric oxide analyzer and normalized against heme and were significantly elevated to ∼6 µM/heme mM at 12.5 h post infective blood meal [55]. These authors suggested that, in contrast to the situation in the mammalian host, haemoglobin drives the production of nitric oxide metabolites in the reducing environment of the blood meal [24]. NO can react with haemoglobin forming a variety of complexes including oxygen radicals and nitroxyl that are also implicated in apoptosis induction. It would seem that once infected blood enters the midgut, conditions there give rise to the generation of additional NO within the blood bolus.

In addition to NO and RNS imbibed with the blood meal, the mosquito midgut cells are themselves a source of NO. Blood meals containing *P. berghei* induce up-regulation of *NOS* expression in the midgut of *An. gambiae* [25] and *An. stephensi* [26]. This *NOS* activity has a biphasic induction in *An. stephensi* with a peak 6 h post-infection, when parasites are developing in the midgut lumen, and at 24–36 h, after ookinete invasion of the midgut [41]. Interestingly, *P. falciparum* glycosylphosphatidylinositols (GPIs), that would be released into the blood meal by both sexual and asexual stages of the parasite, have been shown to induce *AsNOS* expression *in vivo* and *in vitro*, with signaling likely to be mediated by an insulin signal transduction-like pathway [56]. The malaria pigment, haemazoin, has also been reported to induce *NOS* gene expression at 24 h post-feeding, thus producing complimentary activity to GPIs [57]. An additional signal, ingested by the mosquito and activated in the midgut, is mammalian latent transforming growth factor (TFG-β1). Acting as a cytokine, it is a potent regulator of *AsNOS* expression with low doses resulting in sustained induction of anti-parasitic *AsNOS* [41]. Finally, a TFG-β homologue, *As*60A, is inducibly expressed in the mosquito in a parasite density-dependent manner. This also induces NO production and acts to control parasite development [27]. Thus many factors contribute to the toxic, apoptosis-inducing environment in which ookinetes are developing *in vivo*.

Early activity of NOS has been shown to limit parasite development *in vivo* and supplementation of the blood meal with the NOS substrate, L-arginine, significantly reduces the number of oocyst on the midgut of *P. berghei*-infected *An. stephensi.* Furthermore, feeding the NOS inhibitor, L-NAME, increases parasite burden whilst D-NAME, the inactive isomer, has no effect [26] and Cao and co-workers [38] reported a similar effect when mosquitoes were fed on mice that had previously fed on L-NAME or D-NAME. These data do not, however, tell us whether NO/RNS are acting by inducing apoptosis in the parasites.

In this study, feeding mosquitoes blood meals mixed with an NO donor inhibited the development of ookinetes to such a marked degree that hardly any were available to be examined for markers of apoptosis thus the experiments reported here were not able to demonstrate that the death of ookinetes induced by the addition of a NO donor to the blood meal was by apoptosis. We estimate that the concentration of nitrite produced in the midgut by SNAP (the donor used in this experiment) would have been approximately 10 µM. As gametocytes are highly susceptible to the toxic effects of NO and Al-Olayan and co-workers [16] reported signs of apoptosis in the early zygotic stages, midguts containing sporogonic stages clearly need to be examined very shortly after feeding with NO donors to detect the affects *in vivo*. We did however demonstrate that addition of the NOS-activity inhibitor, L-NAME, to the infective blood meal significantly decreased the proportion of ookinetes expressing two markers of apoptosis and increased the number of parasites that transformed into oocysts *in vivo*. This is strong supportive evidence that NO/RNS generated within the mosquito are inducing ookinete death by apoptosis within the midgut lumen, although we acknowledge that NOS activity in WBSs in the blood bolus would also have been inhibited.

### Sources of reactive oxygen species

Peterson et al. [24] suggest that haem and haematin, released within the mosquito midgut during haemoglobin digestion, could react with iron and oxygen in the blood bolus to form ROS. These toxic metabolites are also produced by the mosquito. Lanz-Mendoza and colleagues [40] found that O^•^_2_^–^ is produced naturally in the haemolymph and midgut of *An. albimanus* and is toxic to ookinetes. Additionally, Kumar and colleagues [43] reported *P. berghei*-induced expression of peroxidase genes in the midgut of *An. stephensi*, one of which could produce superoxide anion, lead to the accumulation of high levels of hydrogen peroxide and Molina-Cruz et al [58] have shown that midgut catalase expression is suppressed during midgut traversal and suggest that this leads to high local levels of hydrogen peroxide that contribute to limiting *Plasmodium* infection. The death of ookinetes *in vitro* following combined treatments with SNP and L-DOPA was attributed to the toxicity of peroxynitrite which was formed from the combination of NO produced from SNP and ^•^O_2_^–^ produced from L-DOPA [28]. *In vivo*, ookinetes are developing in a complex redox-active environment (see [24] for a full discussion) and they are thus likely to be exposed to considerable oxidative stress, to which they are highly vulnerable.

### Possible pathways of induction of apoptosis

Following initiation of apoptosis in mammalian cells, two pathways lead to its execution; the intrinsic or mitochondrial dependent pathway and the extrinsic pathway; with considerable cross-talk existing between the two [59]. The former can be initiated by the opening of the inner membrane permeability transition pore or the permeabilization of the outer mitochondrial membrane and release of inter membrane space molecules such as cytochrome c, and Ca^+^. The latter mechanism is mediated by apoptosis regulators, notably in the Bcl-2 family [60], [61].

A mitochondrial dependent pathway had been observed in several parasitic protozoans For example, in *T. cruzi* complement activation leads to an influx of Ca^+^, followed by MOMP loss, increase in oxygen radical production, a decrease in the mitochondrial cytochrome c content and initiation of apoptosis [46]. Although members of the Bcl2 family have not been identified in protozoans, homologues of the mitochondrial nuclease, endonuclease G (EndoG), have been described in yeast, *T. brucei*, *L. donovani* and *L. infantum* [62]–[64]. Additionally a recently described caspase-independent pathway, induced by the presence of a death stimulus, the drug edelfosine, involves the translocation of EndoG straight from the mitochondria of *L. infantum* to the nucleus. Here it is thought to be involved in DNA degradation [63].

Our findings lend support to the hypothesis that a mitochondrial dependent apoptotic pathway exists in *Plasmodium* [15], [18], but suggest that this is not likely to be the only signaling pathway. Examination of MOMP in newly developed ookinetes revealed that a similar proportion exhibited a loss of Δ ψ_m_ as contained pycnotic nuclei. Treatment to induce MOMP loss was 100% successful and also caused a significant increase in the number of parasites with condensed chromatin but, as with other experiments in this study, ∼20% did not exhibit this marker of apoptosis during the period under investigation. This adds more weight to our suggestion that a small proportion of ookinetes may be protected from apoptosis. In contrast to the effect of CCCP incubation, NO did not cause loss of MOMP, giving rise to the conclusion that nitrosative stress-induced apoptosis must operate via an alternative pathway. The induction of apoptosis via nitrosative stress may be attributed to protein nitrosylation [65]. In particular, cysteine proteases could be NO targets via modification of the Cys catalytic residues by S-nitrosylation [66], thus it is possible that NO is activating an apoptosis pathway in the malaria parasite via protein nitrosylation.

Extrinsic pathways leading to apoptosis may involve the proteolytic activation of a family of clan CD cysteine proteases, the caspases [67]. These appear to be confined to metazoans, and may form a secondary executive pathway that evolved after the symbiosis that led to the formation of the eukaryotic cell type. Furthermore, they are not found in all metazoan taxa and it is becoming increasing clear that apoptotic cell death does not always involve these cysteine proteases [68].

Despite the reported involvement of caspase-like molecules in PCD in a variety of single celled organisms (listed in [10]), no caspase homologues had so far been identified in any of their genomes [69]. However, other clan CD cysteine proteases, the metacaspases, have been described in several protozoan [70]. They appear to play a role in some apoptosis pathways in yeast (reviewed in [71]) and may be involved in PCD in Trypanosomatids, *Cryptosporidium parvum* and *Tetrahymena thermophila* [72]–[75]. Although metacaspases have been described in *P. falciparum* [15] and *P. berghei* [76] there is, as yet, no evidence that this is the caspase-like molecule that is involved in apoptosis in *P. berghei* [76] (Arambage and Hurd, unpublished) and clan CA cysteine protease have recently been implicated in programmed cell death in *P. falciparum* [77]. Indeed, the role that metacaspases play in apoptosis in plants, fungi and protozoans is now being debated; they may have additional or alternative functions [78]–[80] and the identity of the caspase-like molecule(s) associated with apoptosis in *P. berghei* and many other protozoans still remains to be determined.

### Conclusions

The majority of ookinetes that form in the mosquito midgut do not succeed in transforming into viable oocysts. Parasite-killing mechanisms such as lysis and melanization operate once the ookinete has traversed the midgut epithelium and attention to date has largely focused on mosquito immune responses that operate at this time. Unless specific markers for apoptosis are used, the majority of ookinetes present in the midgut lumen appear, by light microscopy, to be viable. We suggest that they are not; many of them are already dying by apoptosis and will never invade the midgut. Furthermore NO, RNS and ROS may play a significant role as refractory mechanisms in the redox-active midgut lumen and provide the first line of defence against *P. berghei*.

It is still necessary to determine whether apoptotic death of ookinetes is also an important mechanism for limiting natural mosquito-infections of human parasites such as *P. falciparum*. We have previously shown that *P. falciparum* ookinetes do undergo apoptosis in the midgut of *An. stephensi* in the laboratory [18]. However, the importance of NOS activity in inducing apoptosis, and limiting these infections in the field, is still to be determined and conflicting evidence exists as to the response of *NOS* to natural *Plasmodium* infections in *An. gambiae* [81], [82]. If it does prove to be important, we suggest that it may be feasible to enhance the activity of *NOS*, using transgenic technology, in order to induce apoptosis in most, or all, of the ookinetes before they can traverse the midgut epithelium. We would not anticipate creating undue fitness affects by this strategy because the presence of extra NO, caused by feeding a NO donor with the blood meal, did not affect mosquito longevity, at least at the concentrations of nitrite generated in our experiments (which, in the case of 1 mM SNP is estimated to be approximately 10 µM and thus roughly equivalent to the concentration produced by SNAP that significantly reduced prevalence and intensity of infection). In addition, it may be possible to circumvent some of the loss of reproductive fitness that occurs when mosquito immune responses are up-regulated due to infection if all parasites die very early post-infection and are unable to invade the mosquito midgut [83], [84]. Enhancing the natural apoptotic death of ookinetes in the mosquito midgut lumen could be an important transmission blocking strategy.

Experimental evidence available to date does not supply clues as the evolutionary history or significance of the existence of an apoptosis phenotype in malaria ookinetes. We can speculate that it represents an adaptive, density control strategy that may limit the adverse effects of high infection intensities in the vector and benefit other members of the clone [41]. This could operate via the triggering of mosquito NOS activity in a density dependent way. Equally, density dependent triggers occurring in the mammalian host could be operative in the blood bolus in the midgut lumen. Alternatively, our view of apoptosis in protists may be coloured by knowledge of its functions in metazoans and we may be observing an unavoidable consequence of ookinetes being under oxidative stress or containing defective biochemical pathways and having passed the point of rescue. The latter implying that apoptosis was a mechanism that evolved to be used altruistically in multicellular organisms.

## Materials and Methods

Products were purchased from Sigma-Aldrich Company Ltd. (Dorset, England, UK), unless stated otherwise.

### Ethics statement

All animals were handled in strict accordance with good animal practice as defined by the UK Animal (Scientific Procedures) Act 1986, and all animal work was approved by the UK Home Office, license number PPL 40/2997, and was also approved by the University of Keele Animal Care and Ethical Review Committee.

### Development of ookinetes *in vitro*

*Plasmodium berghei* ANKA 2.34 was maintained in Charles River-Derived (CD1) mice by serial passage for a maximum of 8 passages. Mice were pre-treated by interperitoneal injection of phenylhydrazine two days prior to infection and thick and thin blood smears checked for the presence of infected red blood cells and exflagellating gametocytes from day 3 post infection. Blood was collected by cardiac puncture into a heparinized syringe when parasitaemia reached 5–15% and gametocytaemia was approximately 1–2%. Blood was immediately placed in T25 culture flasks and incubated for 16–19 h at 19°C in RPMI 1604 medium, supplemented with 0.4 mM hypozanthine, 24 mM sodium bicarbonate, 10,000 U penicillin and 10 mg streptomycin/l and 10% foetal calf serum (ookinete medium), as described previously [85], to allow the development of ookinetes. In the majority of experiments, ookinetes were separated from blood cells using MidiMacs (Miltenyi Biotec, Germany) magnetic columns [86], pelleted and resuspended in RPMI-1640 medium or Schneider's medium, before being subjected to different assays. For studies of loss of mitochondrial membrane potential, red blood cells were instead lysed by incubation on ice with 170 mM NH_4_Cl. Ookinetes were pelleted by centrifugation at 800 g for 10 min and ghost red blood cells removed with the supernatant.

### Removal of white blood cells from infected blood

To determine the effect of removal of white blood cells (WBCs) from infected blood prior to incubation, blood was collected from infected mice and one half passed through a CF11 column (Whatman, England) [38] to remove WBCs. Infected blood, with or without WBCs was incubated in ookinete culture medium for 16 h at 19°C, and then ookinetes were collected and further incubated in Schneider's insect medium for 0.25, 3 and 6 h before examining for markers of apoptosis.

### Assays for markers of apoptosis

Morphological markers for apoptosis were detected using slight adaptations of techniques applicable for metazoans, as described in [18]. Briefly, ookinetes exhibiting nuclear chromatin condensation were detected using acridine orange at a working concentration of 2.5 µg/ml. The nuclei of healthy ookinetes remained unstained whereas nuclei with condensed chromatin stained green and nuclear fragmentation was occasionally visible.

Activated caspases-like molecules that have been associated with PCD in *P. berghei* ookinetes were detected using the CaspaTag fluorescent caspase activity kit (Chemicon International, USA) as described in [16], [18]. This contains the pancaspase inhibitor carboxyfluorescein -Val-Ala-Asp- fluoromethyl ketone (FAM.VAD.fmk). PI was used to exclude cells with compromised membranes.

Translocation of phosphatidylserine to the outer surface of the cell membrane was detected with annexin-V FITC apoptosis detection kit according to manufacturer's instructions but incubation took place at 19°C. Ookinetes with compromised membranes were detected using PI. Ookinetes labeled with a green band around the plasma membrane and no PI staining were considered positive whereas those with both annexin and PI labeling were discounted because annexin could have penetrated the cell and stained PS that had not translocated. Thus late apoptosis could not be distinguished from death by non-apoptotic means (see [18] for a discussion of annexin staining and signs of early or late apoptosis).

Loss of mitochondrial trans-membrane potential was detected using a MitoProbeTM JC-I assay kit (Molecular probes, UK) according to manufacturer's instructions except that incubation with JC-1 was carried out at 19°C [18]. This kit included the mitochondrial membrane potential disrupter, CCCP, which was used to create loss of MOMP in ookinetes. In appropriate experiments, one group of ookinetes was incubated with CCCP for 30 min prior to examination for MOMP loss.

Fluorochrome markers of apoptosis were detected using a Leica DM IRB inverted fluorescence microscope under oil immersion (x1000) and recorded on Leica FW 4000 image software (Leica Microsystems, Germany).

### Viability assays

Cells with compromised membranes were detected by staining with either 0.1% erythrosin-B, which stained cells pink (visible under white light), or 250 µM PI.

### The effect of nitric oxide on ookinetes *in vitro*

In order to determine whether exposure to NO induces the display of markers of apoptosis, ookinetes were incubated with the NO donor SNP following elution from a magnetic column. SNP was added to ookinete medium at different concentrations ranging from 10 µM to 4 mM and ookinetes incubated at 19°C for periods of 15 min to 24 h. Following incubation, ookinetes were checked for signs of condensed chromatin, caspase-like activity, translocation of phosphatidylserine to the outer plasma membrane, loss of mitochondrial membrane potential or loss of plasma membrane integrity. The proportion of ookinetes displaying these markers was compared with those incubated in RPMI or Schneider's medium alone. Investigations were conducted in a similar manner using two other donors, SNOG and SNAP to demonstrate that induction of apoptosis markers could occur with other NO donors.

### NO released by NO donors

The Griess reaction [87] was used to make a comparison of the production of NO, in the form of nitrite, by SNP, SNAP and SNOG. Donors were dissolved in supplemented RPMI-1640 (without phenol red) at concentrations ranging from 10 µM to 4 mM and incubated at 19°C for various periods up to 24 h. Nitrite production was determined using a standard curve made from serial dilution of sodium nitrite (range 200–3.125 µM).

### The effect of reactive oxygen species (ROS) on ookinetes in vitro

Two sources were used to provide reactive oxygen species in the RPMI incubation medium; 3, 4-dihydroxy-L-phenylalanin (L-DOPA) and hydrogen peroxide (H_2_O_2_) at different concentrations ranging from 10 µM to 1 mM. Ookinetes were incubated at 19°C with or without these compounds for 1 h or 4 h and markers for apoptosis-like cell death monitored.

### The effect of nitric oxide synthase activity on ookinetes in vivo

Three to six day-old female *An. stephensi* (Dubai) were starved overnight before blood feeding. Three groups of females were fed gametocytaemic mouse blood via an artificial membrane feeder (Haemotek Membrane Feeding System). The NOS inhibitor, L-NAME (1 mg/ml) was added to the blood in one feeder, 1 mg/ml of D-NAME, the inactive isomer, to the second feeder and gametocytaemic mouse blood alone was added to the third feeder, as a control. Fully engorged mosquitoes were separated and transferred to cages supplied with 10% glucose solution and maintained at 19–20°C. Midguts from five mosquitoes per group were dissected at 18 h and 20 h post-feeding, the contents stained with an equal volume of acridine orange (25 µg/ml PBS) and immediately observed using fluorescein filters. The percentage of ookinetes with condensed chromatin per midgut was determined in each of 3 experiments and 200 parasites were counted each time. In a further experiment that followed the same protocol, five mosquitoes from each treatment group were dissected at 15, and 20 h post-feeding and 200 ookinetes in the blood meal of each mosquito were examined for caspase-like activity using CaspaTagTM a fluorescein caspase activity kit (Intergen Company) according to manufacturers instructions, except that incubations were performed at 19–20°C. The proportion of ookinetes that had a compromised membrane was monitored by observing PI penetration.

This protocol was repeated with another generation of mosquitoes except that 50 infected mosquitoes were maintained for twelve days post-infection. A record was kept of the number of mosquitoes dying during the experiment. The remaining mosquitoes from each group were dissected, their guts stained with 0.05% mercurichrome to enhance oocyst visualization and the number of oocysts per midgut counted.

### The effect of nitric oxide donors on mosquito fitness

To determine the effect on mosquito longevity of the presence of additional NO in a mosquito midgut during blood feeding, mosquitoes were fed on *P. berghei* -infected blood through an artificial membrane feeder. Three to six day-old mosquitoes from the same generation were either fed on blood from a gametocytaemic mouse containing 100 µM SNP or the infected blood alone. The experimental protocol was repeated with a new generation of mosquitoes, this time with the addition of 1 mM SNP to blood in one of the membrane feeders. Mosquitoes were maintained in standard conditions in the insectary and fed *ad libitum* on 10% glucose supplemented with 0.05% *para-*aminobenzoic acid, and antibiotics (10000 U penicillin and 7 mg streptomycin/l) and deaths recorded daily.

### The effect of nitric oxide donors on *Plasmodium* development in vivo

Mosquitoes were fed via an artificial membrane feeder on blood from a gametocytaemic mouse with or without the addition of SNAP at a final concentration of 100 µM. Mosquitoes were dissected 16–18 h post-blood meal and the number of ookinetes in the midgut lumen, diluted 1∶4 with PBS, was counted. This experiment was repeated five times and the prevalence and intensity of ookinete infection in the NO donor-treated and untreated mosquitoes was compared.

### Statistical analysis

Data were analysed using two-way ANOVA with a general linear model for two parameters, or one-way ANOVA for one parameter. Data were tested for normality using Shapiro-Wilks' *W* or Anderson-Darling normality tests and, where necessary, percentage data were first transformed using arcsine transformation before being submitted to analysis. Follow-up Tukey's paired comparison tests were run when significant differences were identified by ANOVA. Following log transformation of data, a t-test was used to compare means of ookinete prevalence and intensity in mosquitoes fed a blood meal with or without a NO donor. Kaplan-Meier analysis was used to analyse mosquito survivorship. An alpha value was set at 0.05 for all analyses. Analyses were performed using a Minitab 13.1 or Statistica' 99 Edition Package, version 5.5 for Windows (StatSoft Inc., Tulsa: OK, USA).

## Supporting Information

Figure S1 The effect of removal of WBCs from gametocytaemic blood culture on ookinete apoptosis at 0.25, 3 and 6h post collection. Ookinetes showing chromatin condensation were identified by staining with acridine orange. These data represent a mean of three experiments (n = 3) each with two replicates of counts and each count  = 50 ookinetes. Error bars represent SEM. Bars with different letters are significantly different.
(0.08 MB TIF)

Figure S2 The effect of NO donors on the induction of apoptosis-like death in P. berghei ookinetes. Ookinetes were incubated in RPMI with or without the addition of NO donors at different concentrations or for different time periods and then examined for the presence of markers of apoptosis A: The effect of 2mM SNOG on ookinetes, n = 5 with 100–200 ookinetes examined in each experiment. B: The effect of different concentration of SNAP on ookinetes incubated for 1h; n = 3, 75 ookinetes were examined in each experiment. Apoptosis markers examined were condensed nuclear chromatin (A), activated caspase-like molecules (B). Error bars represent standard error of the mean (SEM). Bars with different letters are significantly different. Empty bars represent controls and patterned bars represent treated ookinetes.
(0.19 MB TIF)

Figure S3 Nitrite production by NO donors. The production of nitrite by different concentrations of SNOG (A), SNP (B) and SNAP (C) at different time points after dissolving the nitric oxide donors in RPMI-1640 without phenol red, supplemented as outlined in the text (ookinete medium). Measurements were made using the Griess reaction. Error bars represent SEM of 2 replicates.
(0.19 MB TIF)
