# Supplementary figures and images for: Naturally Occurring Triggers that Induce Apoptosis-Like Programmed Cell Death in Plasmodium berghei Ookinetes
Source: PLoS One. 2010 Sep 9;5(9):e12634. doi: 10.1371/journal.pone.0012634 (PMC2936559; doi:10.1371/journal.pone.0012634)

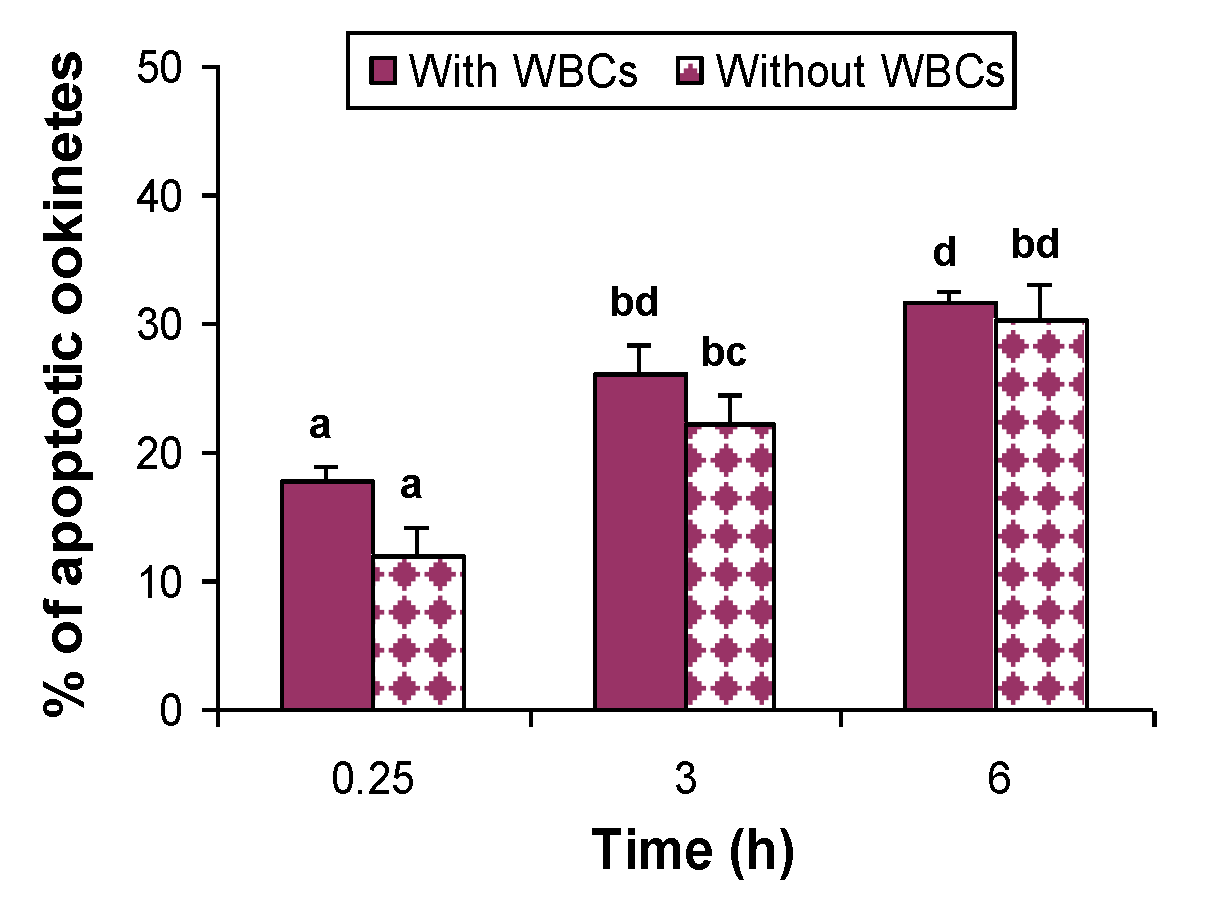

Supplement: Figure S1 — The effect of removal of WBCs from gametocytaemic blood culture on ookinete apoptosis at 0.25, 3 and 6h post collection. Ookinetes showing chromatin condensation were identified by staining with acridine orange. These data represent a mean of three experiments (n = 3) each with two replicates of counts and each count = 50 ookinetes. Error bars represent SEM. Bars with different letters are significantly different. (0.08 MB TIF) [file pone.0012634.s001.tif]

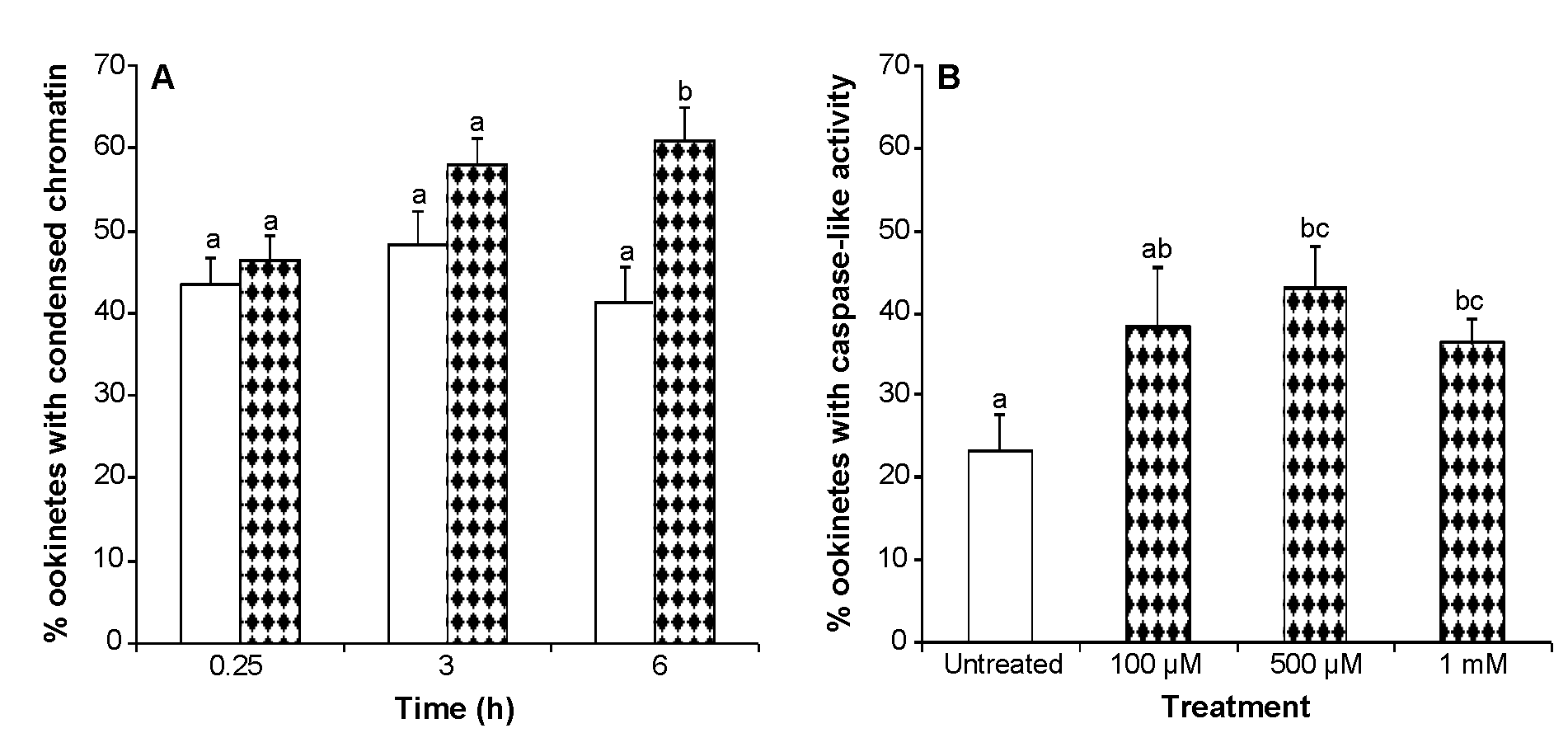

Supplement: Figure S2 — The effect of NO donors on the induction of apoptosis-like death in P. berghei ookinetes. Ookinetes were incubated in RPMI with or without the addition of NO donors at different concentrations or for different time periods and then examined for the presence of markers of apoptosis A: The effect of 2mM SNOG on ookinetes, n = 5 with 100–200 ookinetes examined in each experiment. B: The effect of different concentration of SNAP on ookinetes incubated for 1h; n = 3, 75 ookinetes were examined in each experiment. Apoptosis markers examined were condensed nuclear chromatin (A), activated caspase-like molecules (B). Error bars represent standard error of the mean (SEM). Bars with different letters are significantly different. Empty bars represent controls and patterned bars represent treated ookinetes. (0.19 MB TIF) [file pone.0012634.s002.tif]

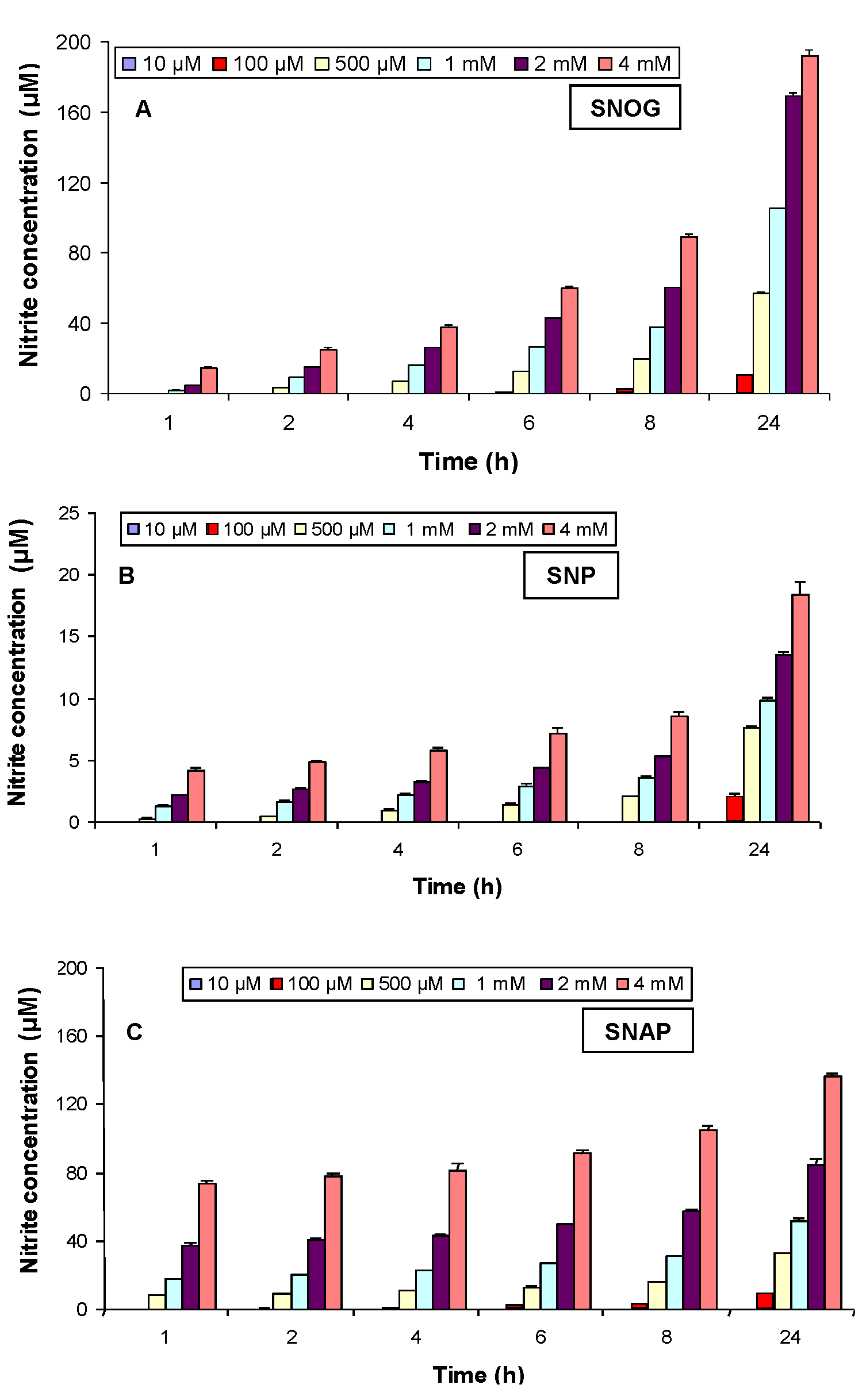

Supplement: Figure S3 — Nitrite production by NO donors. The production of nitrite by different concentrations of SNOG (A), SNP (B) and SNAP (C) at different time points after dissolving the nitric oxide donors in RPMI-1640 without phenol red, supplemented as outlined in the text (ookinete medium). Measurements were made using the Griess reaction. Error bars represent SEM of 2 replicates. (0.19 MB TIF) [file pone.0012634.s003.tif]
